# Radiomics in hepatic metastasis by colorectal cancer

**DOI:** 10.1186/s13027-021-00379-y

**Published:** 2021-06-02

**Authors:** Vincenza Granata, Roberta Fusco, Maria Luisa Barretta, Carmine Picone, Antonio Avallone, Andrea Belli, Renato Patrone, Marilina Ferrante, Diletta Cozzi, Roberta Grassi, Roberto Grassi, Francesco Izzo, Antonella Petrillo

**Affiliations:** 1 Radiology Division, “ISTITUTO NAZIONALE TUMORI – IRCCS - FONDAZIONE G. PASCALE, Napoli, Italy”, Via Mariano Semmola, Naples, Italy; 2 Abdominal Oncology Division, ”ISTITUTO NAZIONALE TUMORI – IRCCS - FONDAZIONE G. PASCALE, NAPOLI, ITALIA”, Via Mariano Semmola, Naples, Italy; 3 Hepatobiliary Surgical Oncology Division, “ISTITUTO NAZIONALE TUMORI – IRCCS - FONDAZIONE G. PASCALE, NAPOLI, ITALIA”, Via Mariano Semmola, Naples, Italy; 4 Division of Radiology, “Università degli Studi della Campania Luigi Vanvitelli”, Naples, Italy; 5 Division of Radiology, “Azienda Ospedaliera Universitaria Careggi”, Florence, Italy; 6 Italian Society of Medical and Interventional Radiology SIRM, SIRM Foundation, Via della Signora 2, 20122 Milan, Italy

**Keywords:** Radiomics, Texture analysis, Liver metastases, Prognosis, Treatment assessment

## Abstract

**Background:**

Radiomics is an emerging field and has a keen interest, especially in the oncology field. The process of a radiomics study consists of lesion segmentation, feature extraction, consistency analysis of features, feature selection, and model building. Manual segmentation is one of the most critical parts of radiomics. It can be time-consuming and suffers from variability in tumor delineation, which leads to the reproducibility problem of calculating parameters and assessing spatial tumor heterogeneity, particularly in large or multiple tumors. Radiomic features provides data on tumor phenotype as well as cancer microenvironment. Radiomics derived parameters, when associated with other pertinent data and correlated with outcomes data, can produce accurate robust evidence based clinical decision support systems. The principal challenge is the optimal collection and integration of diverse multimodal data sources in a quantitative manner that delivers unambiguous clinical predictions that accurately and robustly enable outcome prediction as a function of the impending decisions.

**Methods:**

The search covered the years from January 2010 to January 2021. The inclusion criterion was: clinical study evaluating radiomics of liver colorectal metastases. Exclusion criteria were studies with no sufficient reported data, case report, review or editorial letter.

**Results:**

We recognized 38 studies that assessed radiomics in mCRC from January 2010 to January 2021. Twenty were on different tpics, 5 corresponded to most criteria; 3 are review, or letter to editors; so 10 articles were included.

**Conclusions:**

In colorectal liver metastases radiomics should be a valid tool for the characterization of lesions, in the stratification of patients based on the risk of relapse after surgical treatment and in the prediction of response to chemotherapy treatment.

## Introduction

Radiomics is an emerging field and has a keen interest, especially in the oncology field [1–4]. It has been shown that radiomics could be predictive of TNM grade, histological grade, response to therapy, and survival in various tumors [5–8]. The process of a radiomics study consists of lesion segmentation, feature extraction, consistency analysis of features, feature selection, and model building.

Manual segmentation is one of the most critical parts of radiomics. It can be time-consuming and suffers from variability in tumor delineation, which leads to the reproducibility problem of calculating parameters and assessing spatial tumor heterogeneity, particularly in large or multiple tumors [9, 10].

Radiomic features provides data on tumor phenotype as well as cancer microenvironment. Radiomics derived parameters, when associated with other pertinent data and correlated with outcomes data, can produce accurate robust evidence-based clinical-decision support systems (CDSS) [11]. The potential of radiomics to improve CDSS is beyond doubt and the field is evolving rapidly. The principal challenge is the optimal collection and integration of diverse multimodal data sources in a quantitative manner that delivers unambiguous clinical predictions that accurately and robustly enable outcome prediction as a function of the impending decisions [12]. The central hypothesis of radiomics is that the quantitative individual voxel-based variables are more sensitively associated with various clinical end points compared with the more qualitative radiologic, histo-pathologic, and clinical data more commonly used today [13]. Radiomic variables offer notable advantages over qualitative imaging assessment, since this is clearly limited by the resolution of observers’ eyes. An extension of radiomic information can be accomplished by adding these data to existing prognostic tools, such as genomics. Genomics is an emerging prognostic tool; in fact, genomic markers, along with expression of various microRNA signatures, have been shown to correlate with treatment response, metastatic spread, and prognosis [14–16]. Hence, combining radiomics with genomic data, so-called “radio-genomics,” could potentially provide the highest level of personalized risk stratification ever developed to further advance precision medicine [13]. Radiogenomic may be able to greatly augment patient selection for different cancer therapy, predicting response to treatment, addressing potential resistance to therapy (chemotherapy and/or radiation therapy), distinguishing favorable subsets of malignancies from those with poor prognosis, evaluating which patients may benefit from adjuvant therapy [13, 17].

Hard coded radiomic features were proven to be effective to predict the chemotherapy response in liver colorectal metastases (mCRC), which manifested their clinical usefulness in response prediction [18–20]. However, previously used hard-coded texture features were not specifically designed for targeted clinical issues, which limited their predictive validity. With the development of the deep learning (DL) technique, the neural network is more commonly used in radiomics studies, and has achieved expert-level performance in rectal cancer and liver diseases [21, 22]. DL self-learning quantitative features may supplement unrevealed imaging features besides conventional radiomic features to improve the predictive power. Additionally, DL-based radiomics avoided time-consuming [23].

This article is an update overview on the Radiomics in liver colorectal metastases. Particularly, limitations and future perspectives are discussed.

## Methods

This overview is the result of a self-study without protocol and registration number.

### Search criterion

We assessed several electronic databases: PubMed (US National Library of Medicine, http://www.ncbi.nlm.nih.gov/pubmed), Scopus (Elsevier, http://www.scopus.com/), Web of Science (Thomson Reuters, http://apps.webofknowledge.com/), and Google Scholar (https://scholar.goo-gle.it/). The following search criteria have been used: “Radiomics” AND “Liver colorectal metastases” AND “detection”; “Radiomics” AND “Liver colorectal metastases” AND “diagnosis”; “Radiomics” AND “Liver colorectal metastases” AND **“**Characterization”; “Radiomics” AND “Liver colorectal metastases” AND “Prognosis”; “Radiomics” AND “Liver colorectal metastases” AND “treatment assessment”.

The search covered the years from January 2010 to January 2021. Moreover, the references of the found papers were evaluated for publications not indexed in the electronic database. We analyzed all titles and abstracts. The inclusion criterion was: clinical study evaluating radiomics of liver colorectal metastases. Articles published in the English language from January 2010 to January 2021 were included. Exclusion criteria were studies with no sufficient reported data, case report, review or editorial letter.

## Results

**Fig. 1:**
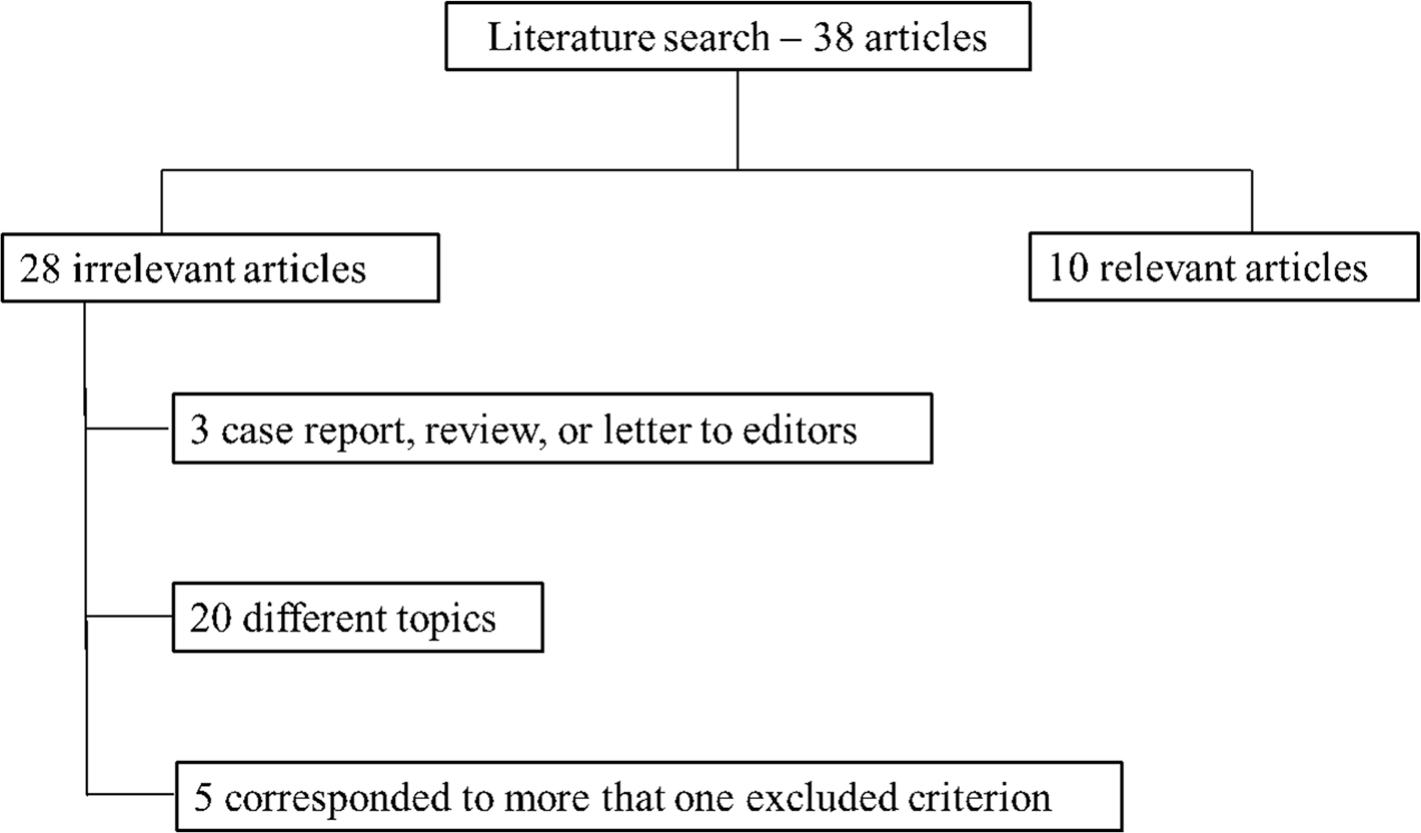
Schematic representation of included and excluded articles

We recognized 38 studies that assessed Radiomics in mCRC from January 2010 to January 2021. Twenty papers were on different topics, 5 corresponds to most criteria; 3 are review, or letter to editors; so 10 articles were included in the analysis (Fig. 1).

## Discussion

### Basic principles and process

**Fig. 2:**
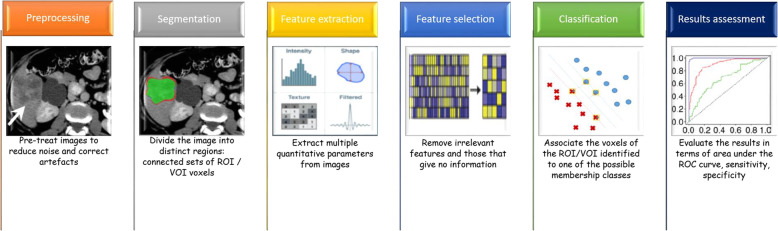
Radiomic Workflow

Radiomics (Fig. 2) is simply the extraction of a high number of quantitative features from medical images, including several phases: image acquisition (e.g. RX, US, MX, CT, MRI, and ibrid imaging as PET-CT or PET-RM); segmentation (aumotatic, semi-automatic and manual); generation of features; development of database; analysis of database and radiomics signature [24]. Images used for radiomic analysis are collected from different hospitals or data centers; thus, these images are usually obtained using different parameters and protocols and reconstructed with different software. The differences may bring unexpected influences on the radiomic model. Segmentation is critical because the subsequent feature data are generated from the segmented volumes. It is challenging because many tumors have indistinct borders. Generation of features refers to the extraction of semantic features such as dimension, necrosis, margin, location or extraction of non semantic features such as shape, hystogram or texture [25–31].

In the past years, texture analysis has gained attention in medical applications and has been proved to be a significant computer-aided diagnostic tool [27]. There is not a strict definition of an image texture but it can be described as the spatial arrangement of patterns that provides the visual appearance of coarseness, randomness, smoothness, etc. Texture analysis describes a wide range of techniques for quantification of gray-level patterns and pixel inter-relationships within an image providing a measure of heterogeneity. It has been shown that different image areas exhibit different textural patterns that are sometimes imperceptible to the human eye [27]. Applications of texture analysis in medical imaging include classification and segmentation of tissues and lesions. Texture analysis applications involve a process that consists of six steps: image acquisition, region of interest (ROI) definition, ROI pre-processing, feature extraction, feature selection, and classification. None of these steps is specific, and the methods have to be chosen according to the application [27]. Manual definition of ROIs is still considered the gold standard in many applications, and it is the chosen option over automatic methods [27]. The size of the ROI should be sufficiently large to capture the texture information thereby eliciting statistical significance. The effect of ROI size becomes insignificant when large ROIs are used. In general, texture features were highly affected at ROI areas smaller than 80 × 80 pixels and became unaffected at ROI areas of around 180 × 180 pixels [27]. It was demonstrated that some features are not only dependent on texture, but also on other ROI properties, such as the mean intensity and variance. To avoid the influence of such factors, ROI normalization is a recommended pre-processing step [32, 33].

**Fig. 3:**
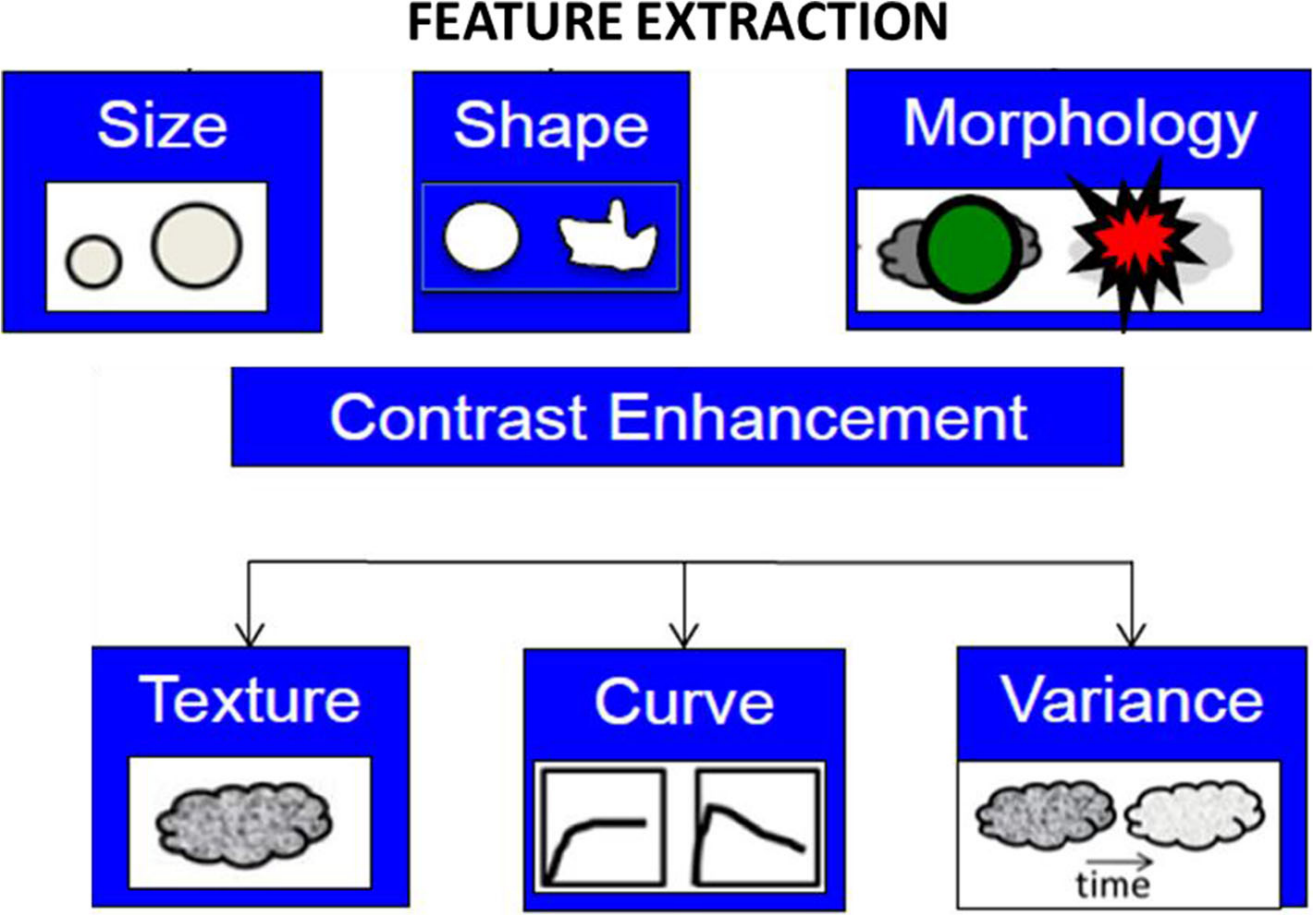
Feature extraction incluing size, shape, morphology features, textural metrics, perfusion parameters

Feature extraction is the main and specific step in the texture analysis process and implies the computation of texture features from predefined ROIs (Fig. 3). Many approaches have been proposed in order to quantify the texture of an image allowing the computation of numerous features, including 2D methods (e.g. Model-based methods, Autoregressive models, Fractal models.) or 3D approaches [34]. 3D approaches increase the dimensionality and the information captured from the image, thus improving the discrimination power [35, 36]. Implementation of 4D texture analysis is possible by including the temporal dimension available in some MRI datasets. Notable results were observed for discrimination of benign and malignant breast lesions and for localization and segmentation of the heart using the 4D spatio-temporal approach [37].

Radiomics can be performed with as few as 100 patients, although larger data sets provide more power. Radiomics could potentially aid cancer detection, diagnosis, assessment of prognosis, prediction of response to treatment, monitoring of disease status [38, 39].

Radiomic is designed to be used in decision support of precision medicine, using standard of care images that are routinely acquired in clinical practice, it presents a cost-effective and highly feasible addition for clinical decision support. Also, this analysis non-invasively characterize the overall tumor accounting for heterogeneity, interrogating the entire tumor allows the expression of microscopic genomic and proteomics patterns in terms of macroscopic image-based features [39]. Moreover, Radiomics Analysis produces prognostic and/or predictive biomarker value derived from routine, standard of care imaging data, allowing for a fast, low-cost, and repeatable means for longitudinal monitoring [39].

## Current applications

### Radiomics approaches in diagnosis and assessment of prognosis

Studies using radiomic analyses have shown that radiomic features are capable of distinguishing between benign and malignant tissue and aiding in the assessment of cancer aggressiveness in a variety of clinical settings [40–43]. Analysis of heterogeneity in enhancement patterns or texture analysis of DCE images has been performed with promising findings; however, texture analysis is not limited to enhancement patterns. Measures of heterogeneity in T1- W, T2W, and DW MRI images can reveal differences in cellular density in tumours, which in turn can be matched to histological findings and aid in distinguishing malignant versus benign lesions [40, 43].

Oyama et al. assessed the accuracy for classification of hepatic tumors by characterization of T1-weighted magnetic resonance (MR) images using two radiomics approaches with machine learning models: texture analysis and topological data analysis using persistent homology**.** They showed that Texture features and persistence images have the potential to capture such arrays of gray scale values and might provide useful information for the differential diagnosis of hepatic tumors [44]. Li et al. assessed the feasibility of texture analysis based on spectral attenuated inversion-recovery (SPAIR) T2W-MRI) for the classification of hepatic hemangioma (HH), hepatic metastases (HM) and hepatocellular carcinoma (HCC). The texture analyses showed that the HH versus HM, HM versus HCC, and HH versus HCC could be differentiated by 9, 16 and 10 feature parameters, respectively. The model’s misclassification rates were 11.7, 9.6 and 9.7% respectively. No texture feature was able to adequately distinguish among the three types of single liver lesions at the same time [45]. Jansen et al. evaluated the MRI data sets of 95 patients with in total 125 benign lesions (40 adenomas, 29 cysts and 56 hemangiomas) and 88 malignant lesions (30 hepatocellular carcinomas (HCC) and 58 metastases). Contrast curve, gray level histogram, and gray level co-occurrence matrix texture features were extracted from the DCE-MR and T2- weighted images. In addition, risk factors including the presence of steatosis, cirrhosis, and a known primary tumor were used as features. Fifty features with the highest ANOVA F- score were selected and fed to an extremely randomized trees classifier. The classifier evaluation was performed using the leave-one-out principle and receiver operating characteristic (ROC) curve analysis. They showed that the overall accuracy for the classification of the five major focal liver lesion types is 0.77. The sensitivity/specificity is 0.80/0.78, 0.93/0.93, 0.84/0.82, 0.73/0.56, and 0.62/0.77 for adenoma, cyst, hemangioma, HCC, and metastasis, respectively [46]. These data are similar to the data by Gatos et al. which reported an overall accuracy of 0.90 for the classification of benign, HCC and metastatic focal liver lesions using only T2-weighted MR images [47].

The prognostic and predictive value of radiomics in colorectal cancer metastases to the liver have been well studied, with several studies demonstrating the utility of diagnostic imaging in predicting clinical outcomes. Data from the current literature highlight the central role of both *KRAS* mutations as strong prognostic and predictive biomarkers among patients undergoing liver colorectal metastases resection (mCRC). In particular, mutations in *KRAS* were strongly associated with worse overall survival (OS) and recurrence-free survival (RFS), as well as specific patterns of recurrence among patients with colorectal liver metastases mCRC [16]. The possibility to correlate radiomic parameters to *KRAS* status offers notable advantages over qualitative imaging assessment, allowing a better patient selection for cancer therapy, predicting response to treatment, distinguishing favorable subsets of patients from those with poor prognosis, evaluating which patients may benefit from surgical treatment. Moreover, a largely underappreciated aspect is the potential that radiomics may be just as pronounced in the economic realm because further optimization of patient selection and early recognition of toxicities undoubtedly influence the cost-effectiveness of cancer care [16]. In our previous study, we assessed the association of RAS mutation status and radiomics-derived data by Contrast Enhanced-Magnetic Resonance Imaging (CE-MRI) in liver metastases. Texture metrics and parameters based on lesion morphology were calculated. Per-patient univariate and multivariate analysis were made. Wilcoxon-Mann-Whitney U test, receiver operating characteristic (ROC) analysis, pattern recognition approaches with features selection approaches were considered. Significant results were obtained for texture features while morphological parameters had not significant results to classify RAS mutation. The results showed that using a univariate analysis was not possible to discriminate accurately the RAS mutation status. Instead, considering a multivariate analysis and classification approaches, a KNN exclusively with texture parameters as predictors reached the best results (AUC of 0.84 and an accuracy of 76.9% with 90.0% of sensitivity and 67.8% of specificity on training set and an accuracy of 87.5% with 91.7% of sensitivity and 83.3% of specificity on external validation cohort) [16]. Also Dercle et al. assessed the role of radiomics signature to predict tumor sensitiveness to irinotecan, 5-fluorouracil, and leucovorin (FOLFIRI) alone (F) or in combination with cetuximab (FC) [48]. The signature (area under the ROC curve [95% confidence interval (CI)]) used temporal decrease in tumor spatial heterogeneity plus boundary infiltration to successfully predict sensitivity to antiepidermal growth factor receptor therapy (FCHQ: 0.80 [95% CI = 0.69 to 0.94], FCSD: 0.72 [95% CI = 0.59 to 0.83]) but failed with chemotherapy (FHQ: 0.59 [95% CI = 0.44 to 0.72], FSD: 0.55 [95% CI = 0.43 to 0.66]). In cetuximab containing sets, radiomics signature outperformed existing biomarkers (KRAS-mutational status, and tumor shrinkage by RECIST 1.1) for detection of treatment sensitivity and was strongly associated with OS (two-sided *P* < .005) [48]. In an analysis of 77 mCRC patients, Lubner et al. investigated tumor heterogeneity through analysis of several quantitative texture parameters, including entropy, skewness, mean positive pixels, and standard deviation [49]. Correlations between these imaging parameters and patients clinico-pathologic features demonstrated an association between less entropy, standard deviation, and a higher mean positive pixel value with tumor grade. Furthermore, the degree of skewness was negatively correlated with KRAS mutations, and entropy was associated with OS [49]. These results were replicated in a retrospective study of 198 preoperative CT scans for patients undergoing resection of colorectal liver metastases [50].

An interesting field of application of radiomics is related to the assessment of lesions before the lesions became detectable. In a pre-clinical study Becker et al. [51] investigate whether any texture features show a correlation with intrahepatic tumor growth before the metastasis is visible to the human eye. Texture analysis was performed on the images yielding 32 texture features derived from histogram, gray level co-occurrence matrix, gray level run length matrix, and gray level size zone matrix. The features were examined with a linear regression model/Pearson correlation test and hierarchical cluster analysis. From each cluster, the feature with the lowest variance was selected. The research showed that texture features may quantitatively detect liver metastases before they become visually detectable by the radiologist.

### Assessment treatment

Radiomics analysis has also demonstrated its potential in assessing treatment response for mCRC patients. In a study of 21 patients, Rao et al. compared CT texture analysis to Response Evaluation Criteria in Solid Tumors (RECIST) for assessing response to chemotherapy. Interestingly, they found that quantitative changes in entropy and uniformity were better at differentiating between good and poor responses when compared to changes in size or volume when using the RECIST criteria [51]. These results suggest that texture analysis may be better at predicting treatment response when compared to conventional size criteria, further expanding the utility of diagnostic imaging. MRI-based texture analysis has also been used to analyze treatment response to Yttrium-90 radioembolization in patients with liver metastases. In a retrospective cohort of 37 patients who underwent radioembolization, serial imaging based upon texture analysis and RECIST criteria monitored treatment response. The researchers showed that in patients with progressive disease, texture analysis was able to detect progression an average of 3.5 months before RECIST [52]. These findings, in addition to the ones previously addressed, demonstrate the diagnostic, prognostic, and therapeutic implications of imaging features, emphasizing their potential to significantly affect liver cancer outcomes [53–58].

#### Current limitations

At present, research on radiomics is still in its infancy and there are no standardized and unified standards for the complicated research process.

Radiomics features contain characteristics of both imaging and numeric features. Radiomics features generally refer to “agnostic” quantitative measurements that are mathematically extracted and differ from “semantic” features such as those covered by radiological lexicons [59, 60]. Four main radiomics phenotypes have been used to capture tissue heterogeneity: 1) volume and shape; 2) first-order.

statistics to assess voxel distributions without considering their spatial relationship; 3) second-order statistics (texture analysis) to study spatial relationships among voxels; and 4) transformed features [61, 62].

Similar to common imaging biomarkers, the reproducibility of radiomics features can be questioned due to the nature of the imaging data itself. For example, intra-individual test-retest repeatability, image-acquisition technique, multi-machine reproducibility, and image reconstruction parameters all contribute in challenging reproducible research in radiomics. Another major challenge is imposed by the variations among the different techniques to process the images into analyzable quantitative data. One can obtain widely different results from the same radiomics data by using different transformation or feature-selection methods. With all these variations in image acquisition and processing in radiomics, it seems a daunting task to obtain a stable, generalizable result that can be consistently reproduced. Therefore, the reproducibility of radiomics features and modeling can be easily challenged, and great effort should be made to reduce variations [63–69].

Although most studies report rigorously quality-controlled manual segmentation, fully automated segmentation algorithms should be implemented for achieving standardization. Another important issue in several studies is the absence of clear identification of causes for false-positive results to further improve the capabilities of Convolutional neural network. In addition, for the selection of ROI, there is currently no suitable algorithm to calibrate tumor regions. Most studies calibrated ROI by radiologists, which increases the amount of pre-work, while calibration by different people will have an impact on the subsequent establishment of the model, leading to limited reproducibility of the results and comparability between studies [65]. In addition, a lack of standardization in reporting the results of research often makes it confusing for readers. We propose that future studies should report features based on the ‘Image biomarker standardisation initiative’ using formal nomenclature and corner marks. Furthermore, traditional machine-learning algorithms such as random forests and deep- learning algorithms like the neural network that have emerged in recent years can both be used for the establishment of radio- mics models. The algorithms used by each type of research are different. Still, there is no research to prove which algorithms are the most suitable for such work. Finally, most of the current research results are still in the training sample stage, so the high accuracy of the model does not reflect its actual predictive ability. Whether the model is effective or not depends on the validation phase by the test sample [65].

Current published studies rely on the data obtained from cohort with limited sample size. These databases should be made with clearly defined criteria, including definite histopathological diagnosis, genomic details, and large amount of clinical and biological data of patients.

## Conclusion

In conclusion, while initial studies looking at radiomics have been very promising, there has been poor standardization and generalization of radiomic results, which limit the translation of this approach into clinical practice. Clear limitations of this field are emerging, especially with regard to data-quality control, repeatability, reproducibility, generalizability of results, and issues related to model overfitting. To address those problems, we propose that future radiomic research should be assessed via the radiomics quality score. By doing so, radiomics studies can be more comparable and increase its potential to be applied in future clinical practice, so that the advance in radiomics will largely contribute to the development of personalization and precision medicine.

## Data Availability

Data sharing not applicable to this article as no datasets were generated or analysed during the current study.
